# New Polylactic Acid Composites Reinforced with Artichoke Fibers

**DOI:** 10.3390/ma8115422

**Published:** 2015-11-16

**Authors:** Luigi Botta, Vincenzo Fiore, Tommaso Scalici, Antonino Valenza, Roberto Scaffaro

**Affiliations:** Dipartimento di Ingegneria Civile, Ambientale, Aerospaziale, dei Materiali, University of Palermo, Viale delle Scienze, Edificio 6, Palermo 90128, Italy; luigi.botta@unipa.it (L.B.); tommaso.scalici01@unipa.it (T.S.); antonino.valenza@unipa.it (A.V.); roberto.scaffaro@unipa.it (R.S.)

**Keywords:** PLA, artichoke fiber, biocomposites, film stacking, quasi-static tensile tests, dynamic mechanical analysis (DMA), scanning electron microscopy (SEM)

## Abstract

In this work, artichoke fibers were used for the first time to prepare poly(lactic acid) (PLA)-based biocomposites. In particular, two PLA/artichoke composites with the same fiber loading (10% *w*/*w*) were prepared by the film-stacking method: the first one (UNID) reinforced with unidirectional long artichoke fibers, the second one (RANDOM) reinforced by randomly-oriented long artichoke fibers. Both composites were mechanically characterized in tensile mode by quasi-static and dynamic mechanical tests. The morphology of the fracture surfaces was analyzed through scanning electron microscopy (SEM). Moreover, a theoretical model, *i.e.*, Hill’s method, was used to fit the experimental Young’s modulus of the biocomposites. The quasi-static tensile tests revealed that the modulus of UNID composites is significantly higher than that of the neat PLA (*i.e.*, ~40%). Moreover, the tensile strength is slightly higher than that of the neat matrix. The other way around, the stiffness of RANDOM composites is not significantly improved, and the tensile strength decreases in comparison to the neat PLA.

## 1. Introduction

Over the last few decades, there has been a growing interest in using new and better performing materials, both in terms of intrinsic properties and from the environmental impact point of view. Nowadays, due to their versatility, composites play a primary role in a wide range of applications and in different fields, such as automotive, aeronautics, civil engineering, architecture and design. Depending on the final application and on the raw materials, the manufacturing process can deeply influence the properties of the final product characteristics [1].

Increasing attention to ecological issues led the researchers to propose natural-based reinforcements with the aim to reduce the environmental impact and to optimize the efficiency of agricultural production, thus reducing disposal problems. In this view, natural fibers are considered as an alternative to conventional synthetic ones as reinforcement for polymer-based composites. In addition to the most common natural fibers (*i.e.*, flax, jute, kenaf, sisal, and so on) [2,3,4,5,6], some authors evaluated the feasibility of using less common fibers, such as *Arundo donax* L. [7,8], okra [9], ferula [10] and althea [11]. In our previous work [12], artichoke fibers extracted from the plant stem were characterized in order to consider them as potential reinforcement of composite structures. The experimental results demonstrated that artichoke fibers showed properties comparable to those of other natural fibers, thus representing a valid alternative. However, no artichoke fiber-reinforced composites were tested to evaluate the real properties of the final material. Nevertheless, composites reinforced with natural fibers often involved conventional oil-based polymers as matrices, with high environmental impact during the production process, service life, life-end and disposal phases. For this reason, matrices obtained from renewable sources were studied by paying particular attention to the material biodegradability. For this purpose, due to its properties, poly(lactic acid) (PLA) represents a good solution in several common applications where traditional polymers are used [13,14,15,16]. Further, the combination of PLA with natural fibers allows one to achieve less expensive materials with increased mechanical properties, useful for semi-structural applications, maintaining the environmental advantage, *i.e.*, compostability.

To the best of our knowledge, no studies on artichoke fiber-reinforced composites are available in the scientific literature. The aim of the present work is to produce PLA-based composites reinforced with artichoke fibers and to evaluate the effect of the fiber placement on the mechanical performances. In particular, random and unidirectional laminates were manufactured by the film-stacking method using PLA matrix. The obtained materials were mechanically characterized by quasi-static tensile tests. Moreover, a dynamic mechanical analysis (DMA) was performed in tensile mode. The morphology of the composites’ fracture surfaces was analyzed through scanning electron microscopy (SEM). Furthermore, a theoretical model, *i.e.*, Hill’s method, was used to fit the experimental Young’s modulus of the biocomposites.

## 2. Experimental Section

### 2.1. Materials

The polymer matrix used to prepare the composites is a sample of PLA (PLA 2002D, Natureworks, Minnetonka, MN, USA, melt flow index (210 °C/2.16 kg) = 6 g/10 min, melting temperature = 151 °C).

Artichoke plants were collected in a plantation in the area of Niscemi (Sicily, Italy). They belonged to the varietal type “Violetto di Sicilia”, one of the seven Sicilian kinds of identified globe artichokes. After collecting the fresh plant, the stem was removed and kept under water for 25 days to allow microbial degradation and, thereafter, fiber extraction. The extracted fibers were then washed with deionized water, dried in open air and then kept in a moisture-proof container.

As reported in our previous work [12], the obtained artichoke fibers have diameters in the range of 100–300 µm and a length between 100 mm and 160 mm. As concerns the thermal stability, their onset degradation temperature is 230 °C. Moreover, their cellulose and lignin contents are equal to 75.3% and 4.3%, respectively.

### 2.2. Composite Preparation

Both PLA and artichoke fibers were dried overnight at 105 °C under vacuum in order to avoid hydrolytic scission of the matrix during processing.

**Figure 1:**
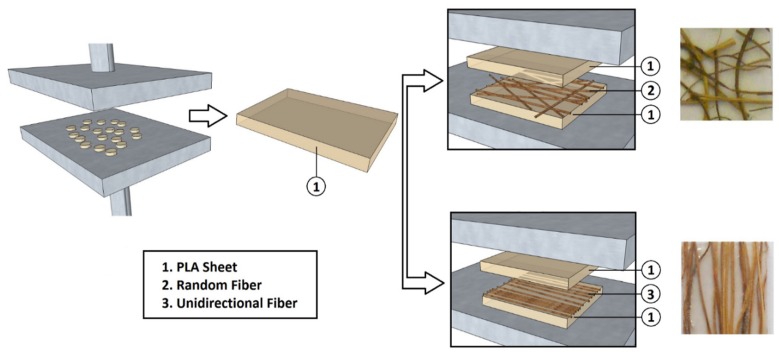
Scheme of the manufacturing process to obtain PLA sheets and PLA/artichoke fiber laminates.

The composites were fabricated using the film-stacking method. As shown in Figure 1, two PLA sheets, previously manufactured by compression molding at 200 °C using a Carver laboratory press (*p* = 25 MPa, time = 3 min) and one fiber hand-sheet were assembled alternately, to obtain a laminate structure. This last one was then placed between two thermo-heated plates of the same laboratory press, pre-heated at 200 °C. A pressure of 25 MPa was applied on the laminate for 3 min. Then, the cold water valve was opened, and the plate temperature was cooled down to 50 °C in about 3 min. At this point, the pressure was released, and the composite panel was removed from the plates.

Two PLA/artichoke laminates were prepared: the first reinforced with unidirectional long artichoke fibers (UNID), the second reinforced by randomly oriented long artichoke fibers (RANDOM). Both laminates were characterized by the same fiber loading (10% *w*/*w*) and thickness (~0.8 mm). For comparison purposes, neat PLA samples were made following the same steps, as well.

### 2.3. Characterization

Quasi-static tensile tests were performed according to the American Society for Testing Material (ASTM) D 638 standard, by using a Zwick-Roell (Ulm, Germany) Universal Testing Machine (UTM), equipped with a load cell of 5 kN. Five dumbbell samples (*i.e.*, dog-bones) were cut off from each laminate by using a Tecno Fustelle die cutter (Torino, Italy). In particular, the width and the length of the narrow section were 6 mm and 33 mm, respectively. The grip distance was 65 mm, and the crosshead speed was set equal to 1 mm/min.

Thirty fibers, dried in the same condition used for the composite preparation (*i.e.*, 105 °C overnight under vacuum), were mechanically tested in tension, according ASTM D 3379, using a UTM by Zwick-Roell, equipped with a load cell of 200 N. The grip distance was 10 mm, and the crosshead speed was set equal to 1 mm/min. The experimental data obtained by mechanical characterization were statistically analyzed using a two-parameter Weibull distribution.

Dynamic mechanical analyses (DMA) were performed in tensile mode, using a MetraVib analyzer Model +150 (Limonest, France). Three prismatic samples of size 44 mm × 5 mm × 0.8 mm were tested for each composite. Temperature scanning was performed from room temperature to 120 °C by setting the heating rate equal to 2 °C/min at an oscillation frequency of 1 Hz, under nitrogen atmosphere.

The morphology of the composites was analyzed by scanning electron microscopy (SEM; Quanta 200 ESEM, FEI, Hillsboro, OR, USA). In particular, the fracture surfaces of dumbbell samples (*i.e.*, those tested under tensile loading) were observed. Before analysis, each sample was sputter coated with a thin layer of gold to avoid electrostatic charging under the electron beam.

## 3. Results and Discussion

### 3.1. Experimental Results

**Figure 2:**
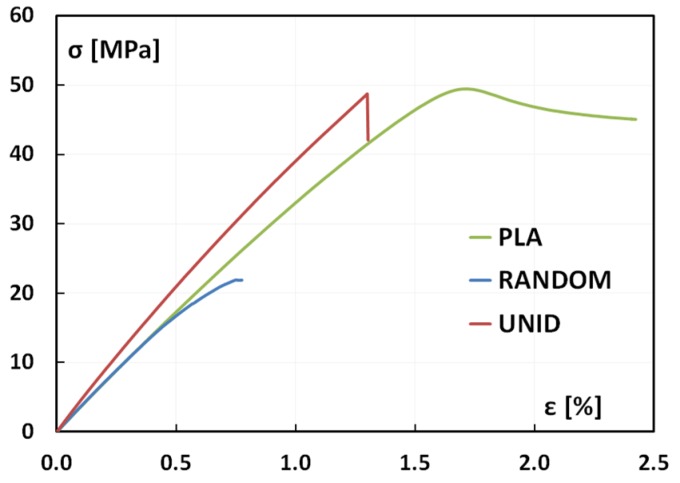
Stress-strain curves obtained from quasi-static tensile tests. UNID, unidirectionally; RANDOM, randomly.

Representative stress-strain curves for each material, shown in Figure 2, clearly evidence different behaviors between neat PLA and artichoke-based composites, although all of the materials present a very low elongation at break. In particular, PLA exhibits necking before fracture; however, such a phenomenon is only partially observed for the RANDOM composites, and it cannot be evidenced for the UNID composites.

Necking is a mode of ductile flow of a material under tension; thus, the stress-strain curves reported in Figure 2 suggest that the addition of artichoke fibers reduces the plastic deformation capability of the matrix, thus leading to a more brittle fracture mode.

**Figure 3:**
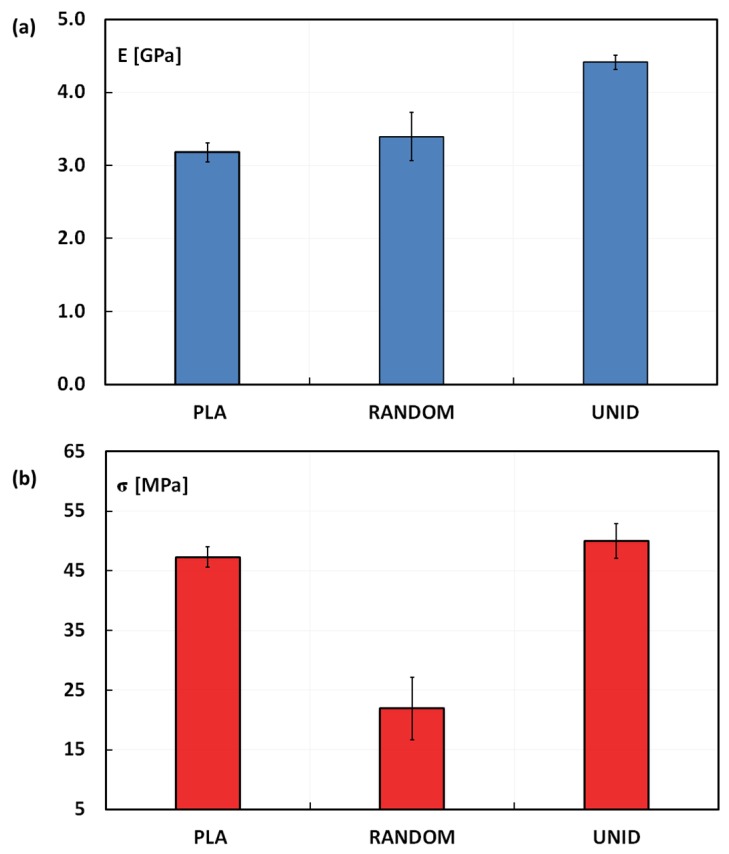
(**a**) Tensile modulus and (**b**) tensile strength of neat PLA and artichoke composites.

The tensile properties of neat PLA and artichoke composites are reported in Figure 3. As shown in Figure 3a, the tensile modulus of UNID composites is significantly higher than that of the neat PLA (*i.e.*, ~40%), even if the fiber content is just 10% by weight. On the other hand, the composite stiffness is not significantly influenced when the artichoke fibers are randomly disposed within the PLA matrix. As expected, the placement of the fibers along the load direction leads to a better reinforcement effect, since this is the best condition to promote the fiber load carrying. On the other hand, no stiffness improvement was found for RANDOM composites, because just a small portion of the fibers is eventually oriented along the load direction. Moreover, even if oriented along the load direction, the specimen manufacturing (*i.e.*, dumbbell shape trimming) does not guarantee the fiber continuity, unlike the UNID composites.

As reported in Figure 3b, the results put into evidence that the UNID composites show an average tensile strength slightly higher than that of neat PLA (50.0 ± 2.9 MPa *vs.* 47.3 ± 1.7 MPa). On the contrary, the RANDOM composites experience a tensile strength drop in comparison to neat PLA (*i.e.*, −53.6%).

In such cases where the fibers mainly bear load (*i.e.*, UNID composites), the prominent damage modes are fiber fracture, some interface debonding, matrix failure and fiber pull-out [17]. In particular, interface debonding and fiber pull-out are typical failure mechanisms mainly due to the weak fiber-matrix adhesion. The morphology of tensile fractured surfaces reflects the different behaviors of composites prepared by disposing the artichoke fibers randomly or unidirectionally oriented within the PLA matrix.

**Figure 4:**
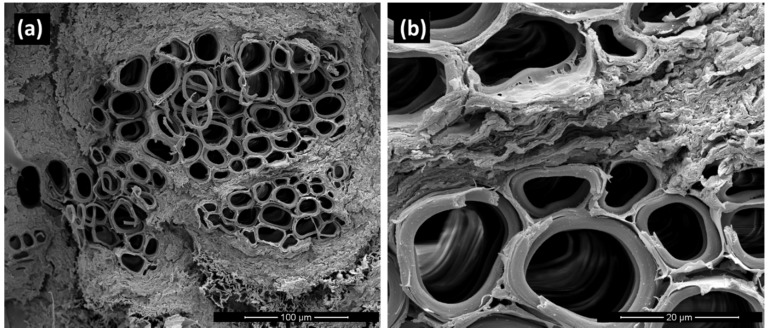
SEM micrographs of the fracture surface of UNID composites at two different magnifications: (**a**) scale bar 100 µm; (**b**) scale bar 20 µm.

Indeed, as evidenced by the micrograph of the fracture surface of UNID composites (Figure 4a), no pull-out damages are visible: *i.e.*, the specimens fail at one cross-section perpendicular to the loading direction, with all of the fibers failing in the same plane.

This can be attributed to the good fiber-matrix adhesion, as clearly shown in the micrograph at higher magnification, reported in Figure 4b. This good adhesion does not allow the fibers to rupture at their weakest location above or below the fracture surface, thus not leading to a lower tensile strength of the composites.

**Figure 5:**
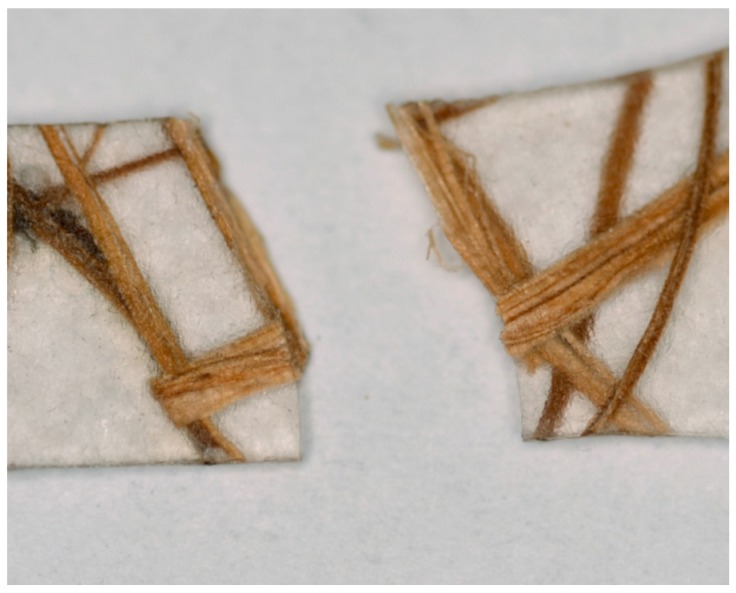
Photograph of a representative fractured RANDOM composite.

As discussed above, although the fiber-matrix adhesion is obviously not dependent on the orientation of the fibers within the matrix, a decrement was found in the tensile strength of RANDOM composites in comparison with that of neat PLA. This behavior can be attributed to the failure mechanism occurring during the tensile tests of RANDOM composites. In particular, it was observed that the failure of RANDOM composite samples always takes place in correspondence to fibers transversely oriented to the loading direction (see Figure 5), which represents the weakest point of the whole specimen. Furthermore, it is evident that the fiber is separated into two portions, each remaining joined to the PLA matrix of the two parts of the fractured sample.

This phenomenon can be explained taking into account the typical structure of the artichoke fibers used in this work. Indeed, as is widely known, natural fibers are packed together, forming what are generally called “technical fibers” or “bundles”, composed of several “elementary fibers” glued together mainly by pectin.

The failure mechanism discussed above indicates that the interface strength between the elementary fibers within bundles is weaker than the fiber/PLA matrix adhesion, as already found for similar systems. In particular, Charlet and Béakou [18] showed that the fiber/fiber interfacial strength within a flax bundle was two- to seven-times lower than those found in the literature for the strength of the interface between flax fibers and classical polymer matrices.

**Figure 6:**
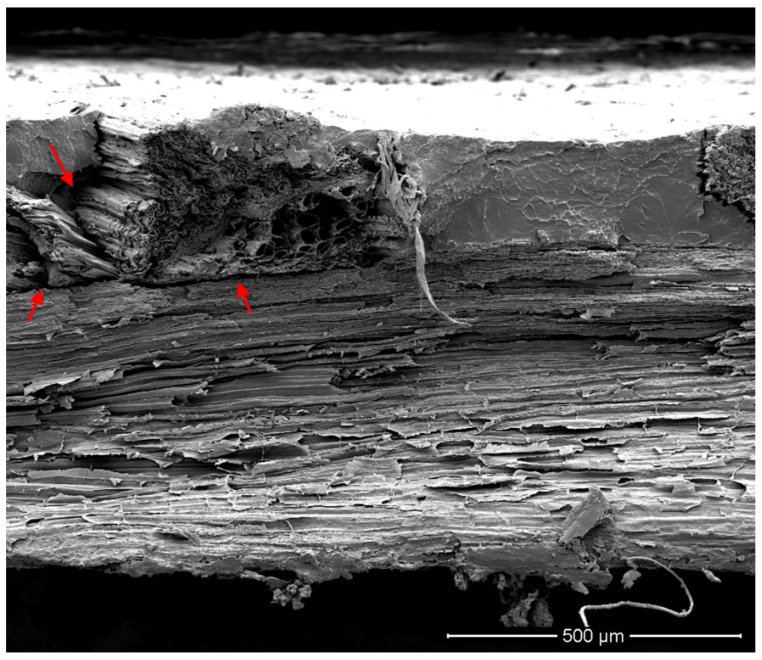
SEM micrographs of the fracture surface of the RANDOM composite. Red arrows indicate matrix-poor areas.

The above observations are corroborated by the SEM micrograph of the fracture surface of RANDOM composites (Figure 6), which shows the fracture surface of the fiber bundle transversally oriented to the loading direction. Failures occur by transverse shearing of the fiber bundles and inter-fiber shearing: *i.e.*, the shear stresses overcome inter-fiber frictional forces, leading to the separation of fiber bundles.

Moreover, the same micrograph evidences that the random placement of the fibers within the PLA matrix can lead to their overlapping. This prevents the PLA from properly wetting the fiber surfaces, thus creating some matrix-poor areas, as indicated by red arrows in Figure 6. Probably, the presence of these voids also contributes to the decrease of the tensile strength of RANDOM composites.

**Figure 7:**
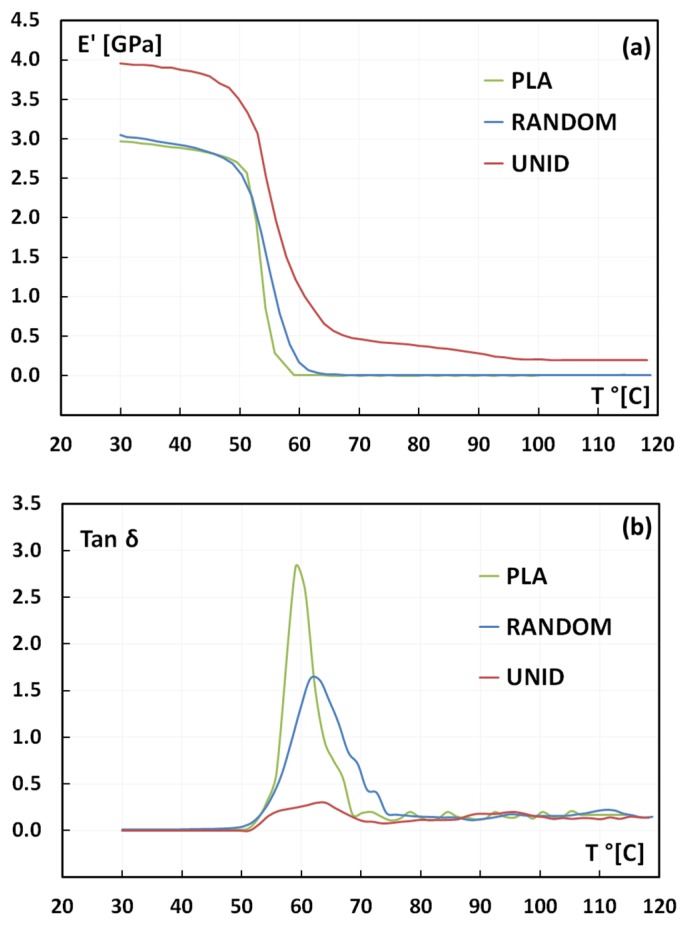
Temperature dependence of (**a**) storage modulus *E*’ and (**b**) tan delta.

As concerns the dynamic mechanical analysis (DMA), the temperature curves of the storage modulus (*E*’) and of the loss factor (tan delta) are shown in Figure 7. The storage modulus of the UNID composites at room temperature is higher than that of the PLA matrix, because of the reinforcement effect of the aligned artichoke fibers (Figure 7a). The other way around, the storage modulus of the RANDOM composites is quite similar to that of neat PLA. These data are in agreement with the static tensile results since, as discussed above: the fibers disposed along the load direction reinforce the PLA matrix better in comparison with the random arrangement.

As shown in Figure 7a, the storage modulus decreases by increasing the temperature for all of the samples, with a significant drop in the range between 50 and 65 °C. Moreover, it is possible to note that the fibers’ placement also influences the softening temperature of the samples. In particular, the drop of E’ occurs at higher temperatures for the UNID composites if compared to the RANDOM composites and the neat PLA, both showing this drop at quite similar temperatures. These results suggest that the unidirectional placement of the fibers implies an extension of the working temperature range of the neat PLA matrix.

Figure 7b shows tan delta factor for neat PLA and its composites as a function of temperature. Incorporation of fibers in a polymeric matrix affects the damping behavior of the composites, which is due both to shear stress concentrations at the fiber-matrix interfaces and to the viscoelastic energy dissipation in the matrix [19,20]. Hence, it depends on the fiber-matrix adhesion: *i.e.*, a weak fiber-matrix adhesion leads to higher values of tan delta [19,21], while a good fiber-matrix adhesion limits the mobility of the polymer chains, thus reducing the damping. In particular, low damping means that the composite has good load-bearing capacity.

As reported in Figure 7b, neat PLA shows a sharp and intense peak, thus indicating no restriction to the macromolecule chain motion. On the contrary, the presence of artichoke fibers hinders the chain mobility, resulting in a broader and larger peak for both the composites, *i.e.*, UNID and RANDOM. This behavior can be attributed to the good fiber-matrix adhesion, as suggested by the tensile results and confirmed by SEM micrographs. More in detail, UNID composites clearly show a broader and lower tan delta peak than RANDOM ones. This different behavior cannot be addressed by changes in fiber-matrix adhesion, which, as already discussed, is not affected by the arrangement of artichoke fibers within the PLA matrix. On the contrary, the different shape of the tan delta peak can be explained taking into account the random placement of the fibers within the PLA matrix. Indeed, this arrangement leads to the overlapping of the fibers, thus creating some matrix-poor areas, as previously evidenced by SEM micrographs. These zones can favor the PLA chain mobility, thus justifying the higher damping shown by RANDOM composites rather than UNID ones.

The glass transition temperature (*T*_g_), evaluated as the temperature at which the damping [19,22] attains its maximum value, is influenced by the presence of artichoke fibers in the PLA matrix. In particular, the *T*_g_ increases from 59 °C to 61.5 °C and 64 °C for RANDOM and UNID composites, respectively. Again, this trend can be associated with the decreased mobility of the matrix chains, due to the presence of the artichoke fibers [23].

### 3.2. Hill’s Method for Tensile Modulus Prediction

Hill’s method was used to calculate Young’s moduli of the PLA composites reinforced with artichoke fibers, with the aim to compare these values with the experimental ones.

Hill’s method refers to the rule of mixtures. In particular, for unidirectionally-reinforced composites, the following equations can be used to evaluate the longitudinal (*E_L_*) and transverse (*E_T_*) moduli, respectively:

(1) $$E_{L}=\nu_{f}E_{f}+\nu_{m}E_{m}$$

(2) $$E_{T}=\frac{E_{f}E_{m}}{\nu_{f}E_{m}+\nu_{m}E_{f}}$$

where *E_f_* and *E_m_* are respectively Young’s moduli of the fiber and matrix; *ν_f_* and *ν_m_* are the volume fractions of the fiber and matrix.

The experimental tensile modulus of PLA matrix (*E_m_*) is equal to 3.18 GPa. The tensile modulus of the artichoke fibers (*E_f_*), determined by performing tensile tests on dried long fibers, is equal to 19.7 GPa. The fiber volume fraction (*ν_f_*), evaluated considering the fiber weigh fraction (*i.e.*, 10% for both composites) and the apparent densities of the PLA matrix and of the artichoke fiber, is equal to 0.081.

For UNID composites, the experimental value of the tensile modulus has to be compared to the theoretical value *E_L_*. On the other hand, Hill’s method allows one to calculate the theoretical Young’s modulus for random fiber-reinforced lamina (*i.e.*, RANDOM composites) as follows:

(3) $$E=\frac{E_{L}+E_{T}}{2}$$

It is possible to demonstrate that *E_L_*(*ν_f_*) represents an upper bound in ideal conditions (*i.e.*, perfect bonding, homogenous fiber and matrix, lack of voids), while *E_T_*(*ν_f_*) is a lower bound.

**Figure 8:**
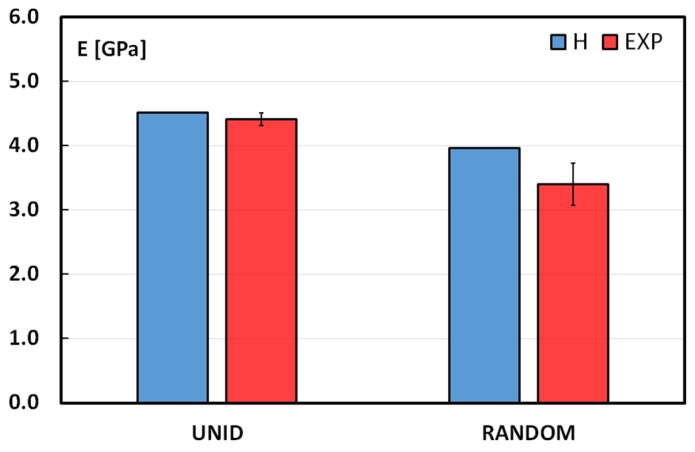
Comparison between Hill (H) and experimental data (EXP) for tensile moduli.

In Figure 8, a comparison between the experimental and theoretical values of Young’s moduli for UNID and RANDOM composites is shown.

**Figure 9:**
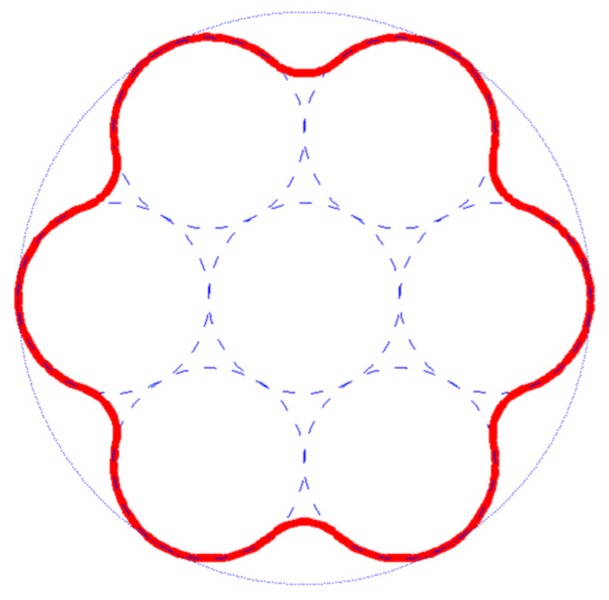
Simplified representation of the cross-section of artichoke fiber.

Regarding the UNID composites, even if the raw materials are far from the ideal conditions, the experimental value is quite close to the theoretical one. This good behavior could be explained taking into account the morphology of the single fiber. A simplified representation of the artichoke fiber cross-section, which shows the packing of several cylindrical elementary fibers in a bundle or technical fiber, is reported in Figure 9.

It is important to note that, taking into account the good fiber/matrix adhesion evidenced by SEM analysis, the bonding surface (highlighted in red in Figure 9) results in being larger than the ideal cylindrical one, thus reducing the fiber-matrix interface shear stresses. This is confirmed by the absence of the pull-out mechanism during tensile tests, as widely discussed in the previous section of this work. On the other hand, the surface shape of a single fiber may promote mechanical interlocking effects, so increasing the shear stress capability of the fiber-matrix interface.

As concerns the RANDOM composites, Hill’s method overestimates the experimental values. This is probably due to the fibers overlapping. As discussed above, this arrangement limits the PLA flow during the composite preparation, so the fiber-matrix stress transfer is not guaranteed in some matrix-poor areas: *i.e.*, artichoke fibers produce a low level of reinforcement.

## 4. Conclusions

The results of this work revealed that new natural fibers, *i.e.*, ones extracted from the stem of the artichoke plant, can be powerfully used as reinforcement for PLA-based composites. It was found that the fiber placement within the matrix (*i.e.*, unidirectionally or randomly oriented) greatly influences the mechanical properties of the composites. In particular, the tensile modulus of UNID composites was significantly higher than that of the PLA (*i.e.*, ~40%), although the fiber loading is just 10% by weight. The other way around, the stiffness of RANDOM composites was not significantly improved. Moreover, the UNID composites showed tensile strength slightly higher than that of the neat matrix, whereas in the RANDOM composites, this property decreased.

The fracture morphology of the samples reflected the different behaviors shown by UNID and RANDOM composites. In particular, no pull-out damages were visible on the fracture surface of UNID composites, due to the good fiber-matrix adhesion. The other way around, the tensile strength decrement experienced by RANDOM composites was attributed to their failure mechanism, *i.e.*, it took place in correspondence to fibers transversely oriented to the loading direction.

The observation, in the dynamic mechanical characterization, of a broader and larger tan delta peak experienced by both of the composites in comparison with the neat matrix, confirmed the good fiber-matrix compatibility.
